# Geriatrics Fellowship-Family Medicine: Evaluation of Fellowship Program Accessibility and Content for Family Medicine Applicants

**DOI:** 10.7759/cureus.10388

**Published:** 2020-09-11

**Authors:** Melissa Chan, Emily Chan, Chapman Wei, Theodore Quan, Chaplin Wei, Jonathan Li, Katalin E Roth

**Affiliations:** 1 Department of Geriatrics and Palliative Medicine, George Washington University School of Medicine and Health Sciences, Washington, USA; 2 Department of Dermatology, George Washington University School of Medicine and Health Sciences, Washington, USA; 3 Orthopaedic Surgery, George Washington University School of Medicine and Health Sciences, Washington, USA

**Keywords:** geriatrics, fellowship content, accessibility

## Abstract

Background: Website content and accessibility has the potential to influence the applicant’s decision whether to interview for the program or not. The objective of our study is to determine the content and accessibility of the American Academy of Family Physicians (AAFP) Directory and accredited geriatric (family medicine) fellowship program websites.

Methods: A list of geriatric (family medicine) fellowship programs was retrieved using the AAFP Directory and verified for accreditation. Contact information was compared between the directory and the fellowship websites. The programs’ website links from the directory were evaluated and compared with Google search. The websites’ accessibility and content were assessed for program, education, and application overview.

Results: Fifty programs were identified, but 43 programs were chosen for analysis. There was an incongruence of over 50% of contact information between the AAFP Directory and the website page. Regarding content, most websites were lacking in fellows’ profile information, previous research studies, and application ID.

Conclusion: AAFP Directory and fellowship websites can improve geriatric (family medicine) fellowship recruitment by updating their information and providing more accessible and accurate content.

## Introduction

About one in five family medicine graduates pursue an additional fellowship, with a significant amount of graduates deciding to pursue geriatrics medicine [1]. Accessibility of fellowship program information is a vital factor for allowing applicants to decide which fellowship program they want to pursue. Most applicants rely extensively on online resources, including online fellowship resources and Google search, to learn more information before applying [2,3]. The American Academy of Family Physicians (AAFP) provides a Fellowship Directory that is an online resource for searching family medicine fellowship content. The objective of our study is to assess the content and accessibility of the AAFP Fellowship Directory and its listed websites in the database.

## Materials and methods

The list of geriatric (family medicine) fellowship programs was retrieved using the AAFP Fellowship Directory. Our study retrieved the following information from the programs’ websites to assess program overview, education plan, and application process [3]. Information to assess program overview included program description, address, phone number, email address, name of program director, description of degree/accreditation, point of contact, email address of point of contact, list of current fellows, list of past fellows, and list of alumni’s work after fellowship. Information to assess the education plan included the presence of didactic sessions, journal club, curriculum schedule, fellow responsibilities, research requirement/opportunity, past research studies, and call description. Information to assess the application process was the presence of ACGME ID and information for the application process. In addition, information that was present in the AAFP Fellowship Directory was cross-referenced with information from the fellowship’s website. Accessibility of content from each geriatric fellowship program in our list was then assessed and compared with Google search of program name followed by ‘geriatric fellowship.’ This paper did not require IRB approval.

## Results

A total number of 50 programs were identified as geriatric fellowship programs in the AAFP Directory. Seven programs were excluded from analysis, as one program was a duplicate and six programs were currently not ACGME-accredited. Thirty-eight programs (88.4%) reported a website link on the directory. Using Google search, all programs (100%) had a functional website link. There was an incongruence of 19 names of program directors (52.8%), 16 phone numbers (55.2%), 22 emails (71.0%), and seven addresses (29.2%) between the AAFP directory and the fellowship website page. When examining the websites, only 13 programs (30.2%) reported names of past fellows, and eight programs (18.6%) reported past fellow’s current work (Table 1). There were a few programs that did not report having didactic sessions, journal clubs, conferences, and research opportunities. Some programs also did not have some form of rotation schedule or clinical duties and responsibilities. Only seven programs (16.3%) reported previous research projects from the program, and seven programs (16.3%) reported about call duties. There were only seven programs (16.3%) that reported their application ID and eight programs (18.6%) did not report any information on the application process.

**Table 1:** Assessment of website fellowship content

Program Characteristics	N (%)
Program Description	42 (97.7%)
Program Director's name	42 (97.7%)
Phone number	30 (69.8%)
Email address	38 (88.3%)
Address	22 (51.1%)
Names of current fellows	23 (53.5%)
Names of past fellows	13 (30.2%)
Past fellow's current work	8 (18.6%)
Other degrees offered	1 (2.3%)
Didactic sessions	31 (72.1%)
Journal club	28 (65.1%)
Rotation schedule	37 (86.0%)
Meetings and conferences	34 (79.1%)
Clinical duties and responsibilities	41 (95.3%)
Research opportunities	40 (93.0%)
Research requirement	33 (76.7%)
Program's previous research projects	7 (16.3%)
Call duties status	7 (16.3%)
Application ID	7 (16.3%)
Application process information	35 (81.4%)

## Discussion

This study determined the accessibility of geriatric fellowship program content for family medicine residents in the AAFP Directory database and fellowship websites. The information in the AAFP Directory and the fellowships’ websites displayed inconsistencies regarding program overview. Studies have shown that residents do in fact use the AAFP Directory Database when conducting their search for fellowship programs [4]. Regarding fellowship website content, there can still be an improvement in its education plan and application process for geriatric fellowship applicants. This study is important as it highlights the need for various information that can help guide an applicant's decision to choose a specific program. Furthermore, accurate and more information on these websites can ease the stress that applicants may have when researching these programs, as all of the information can be centralized on the websites. Petriceks et al. indicated that there needs to be an increase in geriatric fellow-trained physicians because of the increased number of extremely complex older patients [5]. Since other studies have suggested that the improvement of accessibility and content quality of fellowship websites may increase candidate recruitment, updating the AAFP Directory and improving fellowship website content may increase the number of family medicine applicants pursuing a geriatric fellowship [2,6].

## Conclusions

This study identified difficulties and challenges for family medicine applicants trying to access important content and information when considering a geriatric fellowship. There can be improvements to updating more accurate, valuable, and standardized content that is easy for the viewer to navigate so geriatric fellowship programs have an easier time to recruit applicants.
